# Exogenous Melatonin Alleviates High- and Low-Temperature Stress in Cucumber Seedlings and Regulates Hormone Signaling-Related Transcriptional Responses

**DOI:** 10.3390/plants15193066

**Published:** 2026-10-08

**Authors:** Yinxin Yang, Lushan Li, Xuan Wang, Sijie Tang, Zihui Yang, Haijia Gui, Zhenghai Sun, Yiqing Feng

**Affiliations:** Yunnan Key Laboratory of Landscape Plant Resource Cultivation and Application, College of Landscape Architecture and Horticulture Sciences, Southwest Forestry University, Kunming 650224, China; yangyinxin@swfu.edu.cn (Y.Y.); 18086945551@163.com (L.L.); wangxuan@swfu.edu.cn (X.W.); 3488503755@swfu.edu.cn (S.T.); 15987434291@swfu.edu.cn (Z.Y.); guihaijianczz@163.com (H.G.)

**Keywords:** *Cucumis sativus*, temperature stress, melatonin, transcriptome, Hormonal signaling

## Abstract

Temperature stress is a major environmental factor that limits the growth of cucumber (*Cucumis sativus* L.). Melatonin has been implicated in plant responses to abiotic stress; however, its transcriptional effects on cucumber under high- and low-temperature conditions remain poorly understood. In this study, cucumber seedlings were exposed to 25 °C, 40 °C, or 4 °C, with or without exogenous melatonin treatment at 75 μmol·L^−1^, and their transcriptional responses were investigated using RNA sequencing (RNA-seq). Both high- and low-temperature treatments caused varying degrees of seedling wilting, whereas this phenotype was partially alleviated by melatonin application. Transcriptome analysis identified 7488 and 3400 differentially expressed genes in response to high and low temperatures, respectively, indicating both shared and temperature-specific transcriptional responses. Kyoto Encyclopedia of Genes and Genomes (KEGG) enrichment analysis showed that pathways related to plant hormone signal transduction, phenylpropanoid biosynthesis, and protein processing in the endoplasmic reticulum were significantly enriched in multiple comparisons. Further analysis revealed that melatonin treatment altered the expression patterns of several hormone signaling-related genes and transcription factors, including members of the ERF, MYB, WRKY, and bHLH families. Notably, hormone signaling components exhibited distinct transcriptional responses under high- and low-temperature conditions. The expression trends of selected genes determined by quantitative real-time polymerase chain reaction (qRT-PCR) were generally consistent with the RNA-seq results. Collectively, exogenous melatonin application was associated with reduced wilting symptoms and coordinated transcriptional changes in hormone signaling pathways under temperature stress. These findings provide a basis for further elucidating the molecular mechanisms underlying melatonin-mediated temperature adaptation in cucumber seedlings.

## 1. Introduction

Cucumber (*Cucumis sativus* L.) is a climbing herbaceous species belonging to the genus *Cucumis* in the family Cucurbitaceae. It prefers warm and humid conditions and is sensitive to both low temperature and drought. Owing to its crisp texture, distinctive flavor, and high nutritional value, cucumber is widely cultivated worldwide and represents an economically important horticultural crop. As a thermophilic species, cucumber is particularly sensitive to fluctuations in environmental temperature [1]. Both abnormally high and low temperatures can restrict the normal physiological growth of cucumbers and affect their cellular homeostasis, photosynthesis, and the formation of reproductive organs, as well as fruit yield and flesh texture. [2,3,4,5]. Temperature stress has therefore become an important environmental constraint to cucumber production and quality [6,7,8,9]. Low-temperature stress can alter membrane properties and disrupt cellular ion homeostasis, thereby inducing a series of metabolic disturbances [10]. By contrast, high-temperature stress can disturb cellular protein homeostasis and is often accompanied by excessive accumulation of reactive oxygen species (ROS) in plant tissues [11]. Excessive ROS can impair photosynthesis and, when not effectively controlled, cause severe cellular damage or even cell death. In previous studies on cucumber, natural variations in *CsSGR* have been shown to be associated with cold tolerance; inhibition of *CsGPA1* reduces Ca^2+^ influx under low-temperature conditions, thereby regulating the plant’s tolerance to cold stress [7,9]. Genetic analyses of adult cucumber plants have also identified five loci associated with heat tolerance through genome-wide association studies, including gHII3.1, gHII3.2, gHII3.3, gHII4.1, and gHII6.1 [8]. These findings have provided an important basis for understanding temperature-stress responses in cucumber. Collectively, available evidence indicates that although both high and low temperatures represent forms of thermal stress, their perception and downstream regulatory mechanisms are not identical. Comparative analysis of cucumber responses to high and low temperatures within the same genetic background and developmental stage is therefore important for understanding the molecular basis of temperature adaptation.

Plant hormones are central regulators that link environmental signal perception with growth, development, and stress adaptation [12,13]. Auxin (IAA), cytokinins (CKs), gibberellins (GAs), and brassinosteroids (BRs) are primarily involved in cell division, cell elongation, and organ development [14,15], whereas abscisic acid (ABA), ethylene (ET), jasmonic acid (JA), and salicylic acid (SA) are widely involved in plant responses to environmental stresses [16,17,18]. These hormonal pathways also interact through synergistic or antagonistic crosstalk to coordinate plant growth and stress acclimation [19]. Importantly, hormonal responses to high- or low-temperature stress do not follow a simple and universal pattern in which a particular hormone consistently increases or decreases. Instead, the direction and magnitude of hormonal responses depend on the species, tissue type, developmental stage, and stress intensity [19]. Accordingly, integrated analysis of multiple hormone signaling pathways and their downstream regulatory components may provide a more comprehensive understanding of transcriptional regulation under abnormal temperature conditions than analysis of individual hormone levels alone.

Plant growth regulators have been widely used to improve plant performance under adverse environmental conditions [20]. Among them, exogenous melatonin has received increasing attention as a relatively environmentally friendly and economical regulator that can enhance plant tolerance to abiotic stress [21]. Melatonin was once considered a molecule specific to animals but has since been shown to occur widely in plants [22]. In plant cells, melatonin functions through multiple regulatory mechanisms. Previous studies have shown that exogenous melatonin can influence ROS homeostasis, antioxidant defense systems, and photosynthetic performance, although its functions are not limited to direct or indirect redox regulation [22,23]. Increasing evidence also suggests extensive interactions between melatonin and endogenous plant hormone networks [24]. For example, under combined low-temperature and high-humidity stress, exogenous melatonin significantly improved photosynthetic performance and enhanced antioxidant defense in cucumber seedlings [23]. Under combined low-temperature and low-light stress, melatonin not only improved photosynthetic activity in pepper seedlings but also altered processes associated with auxin and cytokinin metabolism [25]. In apple (*Malus domestica*), Gao et al. reported that both overexpression of the melatonin biosynthesis gene *MdASMT9* and exogenous melatonin application enhanced heat tolerance, and that *MdWRKY33*-mediated transcriptional regulation of *MdNCED1* and *MdNCED3* was associated with changes in ABA accumulation [26]. In strawberry, exogenous melatonin has also been linked to changes in the DREB/CBF–COR cold-response pathway and antioxidant defense [27]. These findings suggest that melatonin-mediated stress responses may involve coordinated regulation of redox homeostasis, canonical stress-responsive pathways, and plant hormone signaling networks. However, the specific regulatory patterns appear to vary among plant species and stress conditions [24]. Although increasing numbers of studies have revealed the roles of melatonin in plant adaptation to diverse environmental stresses, studies in cucumber have mainly focused on specific low-temperature conditions or combined environmental stresses [23]. In contrast, comparatively little is known about how melatonin affects transcriptional responses to high and low temperatures within a unified experimental system, particularly with respect to plant hormone signaling networks.

Therefore, in the present study, cucumber seedlings were treated with exogenous melatonin and exposed to high- or low-temperature stress to systematically compare shared and temperature-specific transcriptional responses. Particular attention was given to the association between melatonin treatment and transcriptional changes in plant hormone signaling pathways. The results provide a basis for further understanding the molecular mechanisms by which melatonin participates in cucumber temperature adaptation and may also offer useful information for its potential application in crop stress management.

## 2. Results

**Figure 1:**
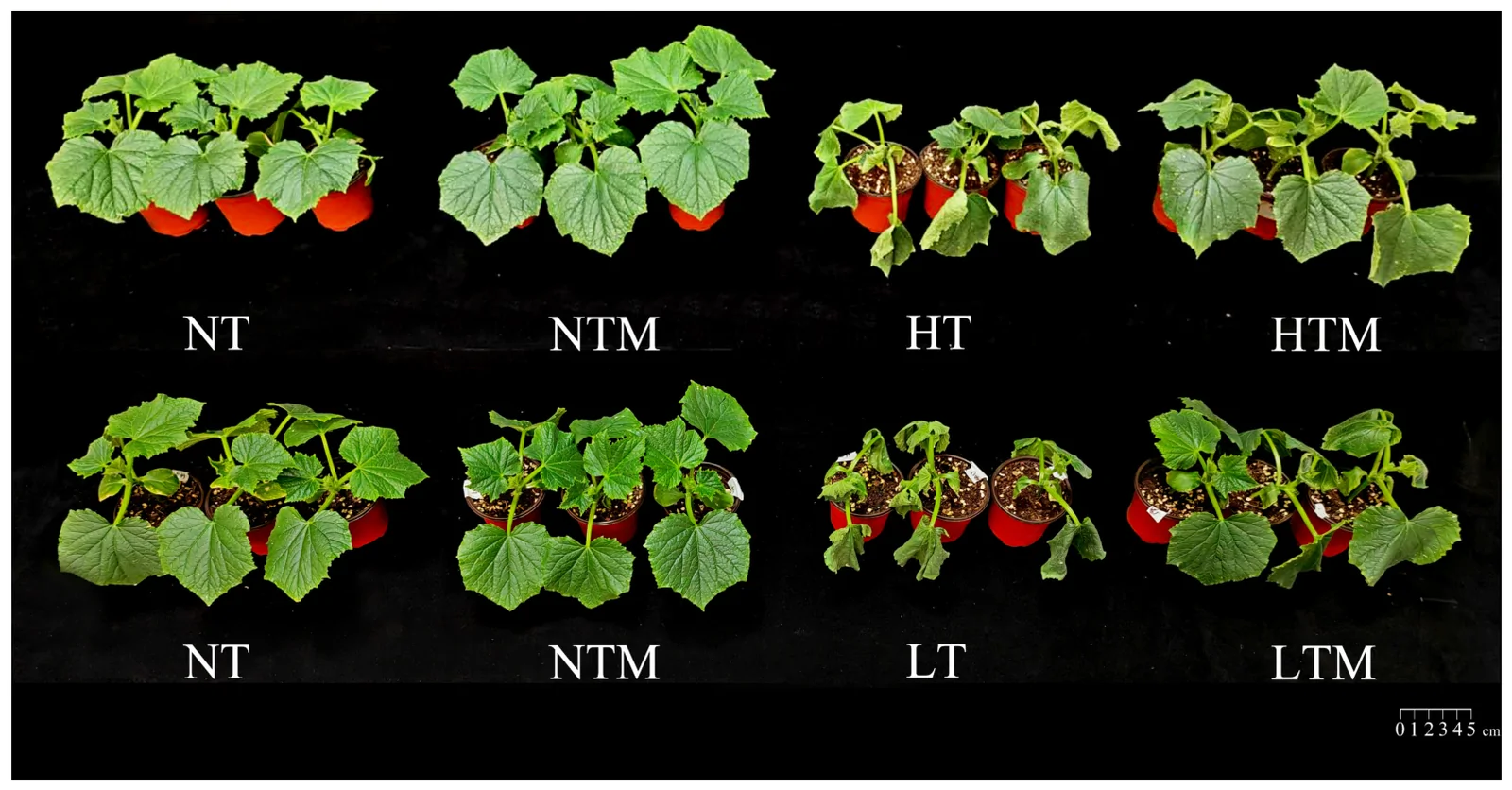
Analysis of phenotypic differences in cucumbers across different experimental groups: NT: treated at 25 °C for 12 h following foliar application of distilled water; HT: treated at 40 °C for 12 h following foliar application of distilled water; LT: treated at 4 °C for 12 h following foliar application of distilled water; NTM: treated at 25 °C for 12 h following foliar application of 75 μmol·L^−1^ melatonin; HTM: 12 h at 40 °C following foliar application of 75 μmol·L^−1^ melatonin; LTM: 12 h at 4 °C following foliar application of 75 μmol·L^−1^ melatonin.

Cucumber seedlings exhibited varying degrees of wilting under both heat and cold stress treatments; following the application of a 75 μmol·L^−1^ melatonin solution, the cucumber seedlings showed some degree of relief from the stress in both heat and cold stress treatments (Figure 1). 

To gain a deeper understanding of the effects of different experimental treatments on cucumbers, we performed transcriptomic sequencing on cucumber samples from various treatments and filtered the sequencing data, yielding a total of 130.67 G of clean data. The amount of valid data per sample ranged from 6.98 to 7.55 G, with Q30 base coverage ranging from 93.51 to 95.66 per cent and an average GC content of 43.55 per cent. By aligning the reads to the reference genome (CuGenDBv2) (accessed 20 June 2026), we obtained the genomic alignment results for each sample, with alignment rates ranging from 97.71 to 98.64 per cent (Table 1). These results indicate that the quality of this transcriptomic sequencing is sufficient for subsequent analysis.

**Figure 2:**
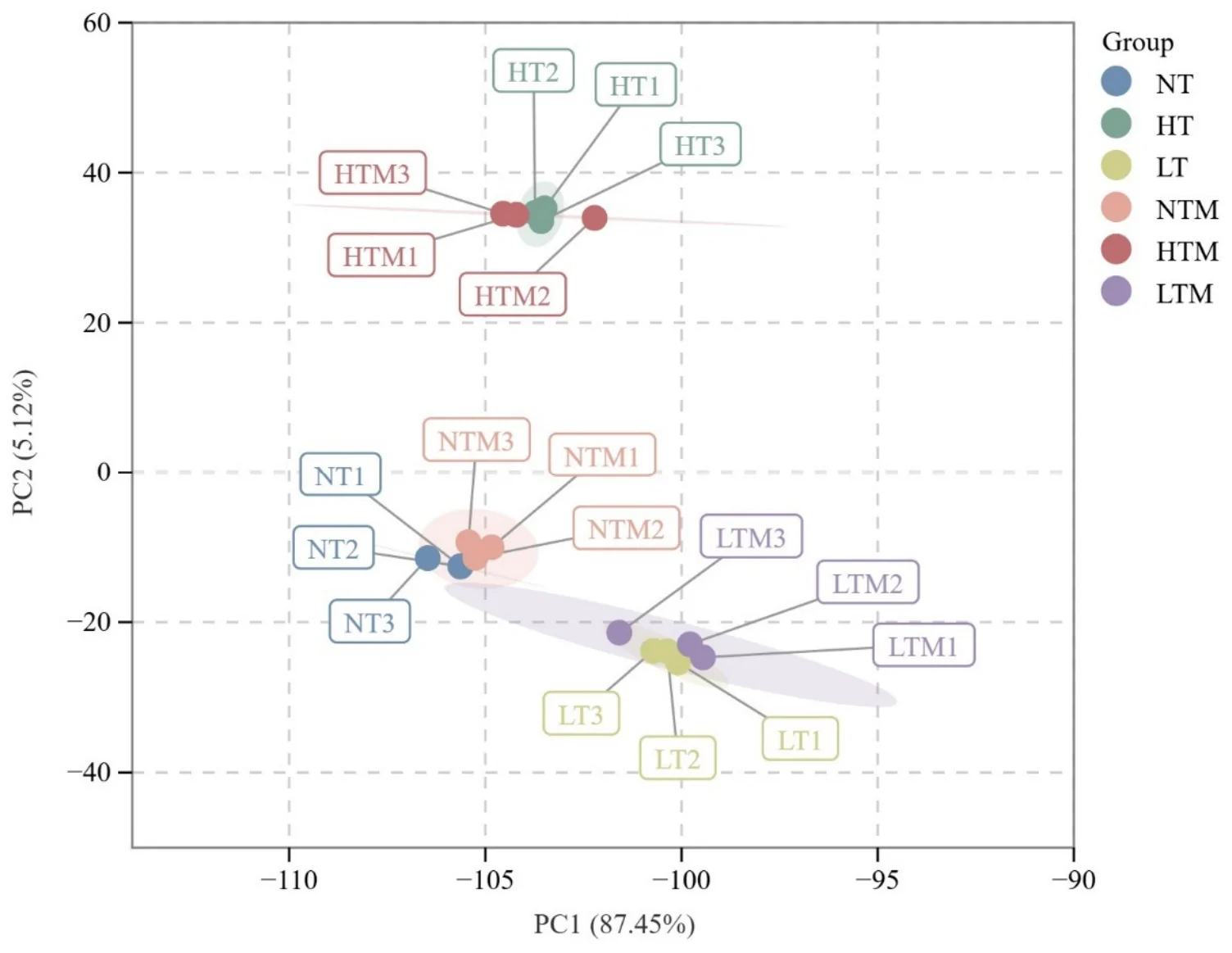
PCA of differentially expressed genes in cucumber leaves across different experimental groups. NT: treated at 25 °C for 12 h following spraying with distilled water; HT: treated at 40 °C for 12 h following spraying with distilled water; LT: treated at 4 °C for 12 h following spraying with distilled water; NTM: treated at 25 °C for 12 h following foliar spraying with 75 μmol·L^−1^ melatonin; HTM: 12-h treatment at 40 °C following foliar application of 75 μmol·L^−1^ melatonin; LTM: 12-h treatment at 4 °C following foliar application of 75 μmol·L^−1^ melatonin.

The set of differentially expressed genes generated was analyzed using principal component analysis (PCA). The results showed that the three replicates from each experimental treatment clustered together, with the biological replicates within each group clustering closely, indicating good consistency among the samples within each group. The large distances observed between the different temperature treatments reflected the significant differences observed under each temperature treatment (Figure 2).

### 2.1. Differentially Expressed Genes in Cucumber Under Temperature Stress

**Figure 3:**
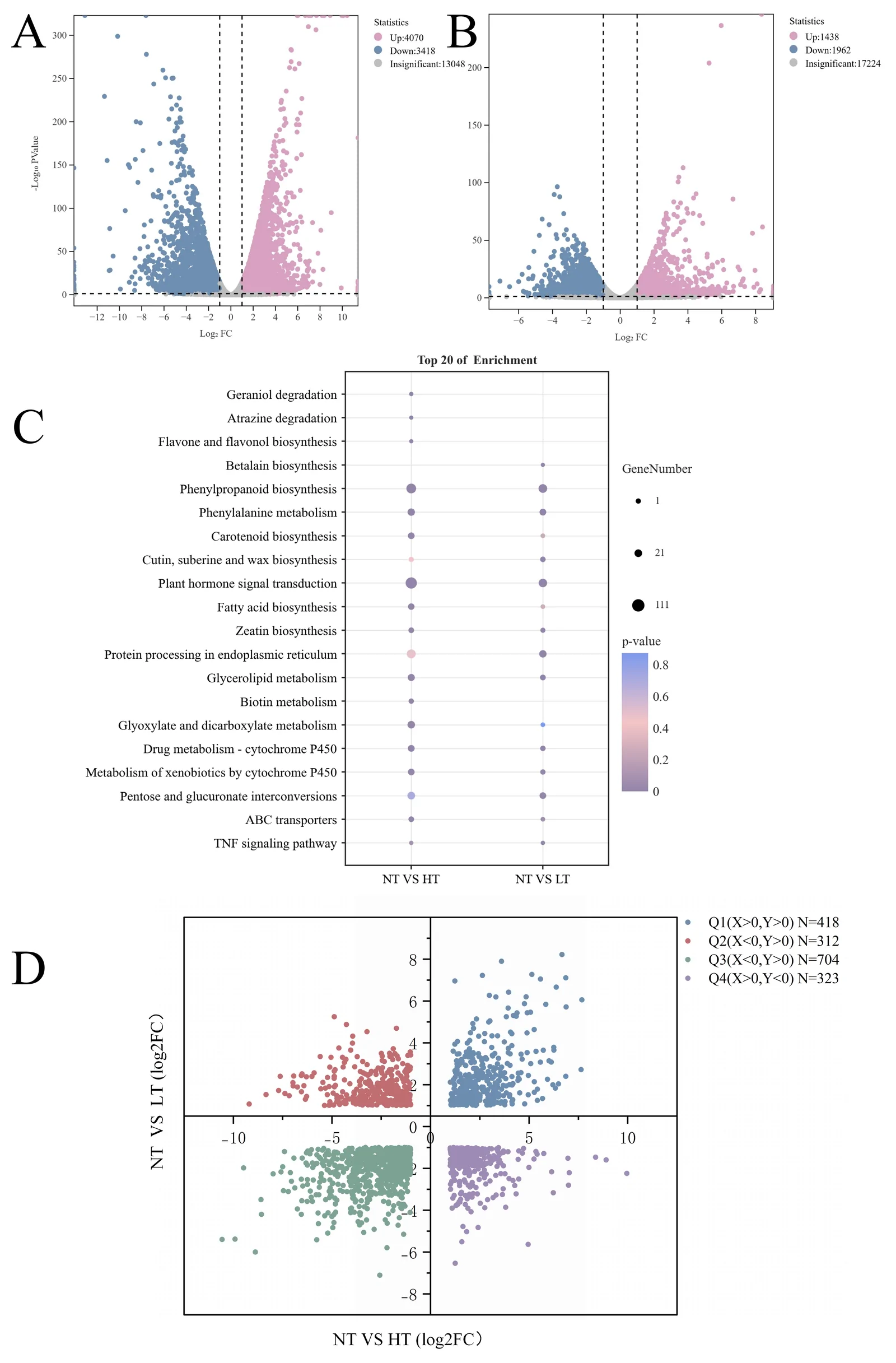
Differentially expressed gene analysis of cucumber under heat and cold stress. NT, cucumber seedlings sprayed with distilled water and maintained at 25 °C for 12 h; HT, seedlings sprayed with distilled water and exposed to 40 °C for 12 h; LT, seedlings sprayed with distilled water and exposed to 4 °C for 12 h. In the volcano plots, each point represents a gene. Blue and pink points indicate significantly downregulated and upregulated genes, respectively, whereas gray points indicate genes without significant differential expression. The x-axis represents the change in gene expression (log_2_FC), with larger absolute log_2_FC values indicating greater differences in relative gene expression between the two groups. DEGs were identified using the thresholds |log_2_FC| > 1 and *p* < 0.05. In the KEGG enrichment bubble plots, the x-axis represents the enrichment factor, calculated as the ratio of the number of DEGs assigned to a given pathway to the total number of genes annotated to that pathway. A larger enrichment factor indicates stronger pathway enrichment. The y-axis shows the KEGG pathway names. Bubble color represents the *p* value, with a more purple color indicating greater statistical significance, whereas bubble size represents the number of DEGs enriched in each pathway. In the comparative analysis of DEGs, the x-axis represents the log_2_FC values under heat stress, and the y-axis represents those under cold stress. Q1 (blue) represents genes upregulated under both heat and cold stress; Q2 (red) represents genes downregulated under heat stress but upregulated under cold stress; Q3 (green) represents genes downregulated under both heat and cold stress; and Q4 (purple) represents genes upregulated under heat stress but downregulated under cold stress. (**A**) Volcano plot of DEGs under heat stress. (**B**) Volcano plot of DEGs under cold stress. (**C**) KEGG enrichment analysis of DEGs under heat and cold stress. (**D**) Comparative analysis of DEGs shared between heat and cold stress.

A total of 7488 differentially expressed genes (DEGs) were identified in cucumber under heat stress, including 4070 upregulated and 3418 downregulated genes (Figure 3A). Under cold stress, 3400 DEGs were detected, of which 1438 were upregulated and 1962 were downregulated (Figure 3B). These results indicate that heat stress induced a broader transcriptional response than cold stress in cucumber.

Kyoto Encyclopedia of Genes and Genomes (KEGG) enrichment analysis showed that Plant hormone signal transduction was the most significantly enriched pathway under both heat and cold stress, followed by Phenylpropanoid biosynthesis and Protein processing in endoplasmic reticulum (Figure 3C). These results suggest that hormone signaling, secondary metabolism, and protein homeostasis are major components of the cucumber transcriptional response to temperature stress.

Comparison of the DEGs induced by heat and cold stress identified 1757 genes that responded to both treatments. Among these shared DEGs, 418 genes were upregulated under both heat and cold stress, whereas 704 genes were downregulated under both conditions. In addition, 312 genes were downregulated by heat stress but upregulated by cold stress, while 323 genes were upregulated by heat stress but downregulated by cold stress (Figure 3D). These distinct expression patterns indicate that cucumber exhibits both shared and stress-specific transcriptional responses to high- and low-temperature conditions.

### 2.2. Differentially Expressed Genes in Cucumbers Following Melatonin Treatment

**Figure 4:**
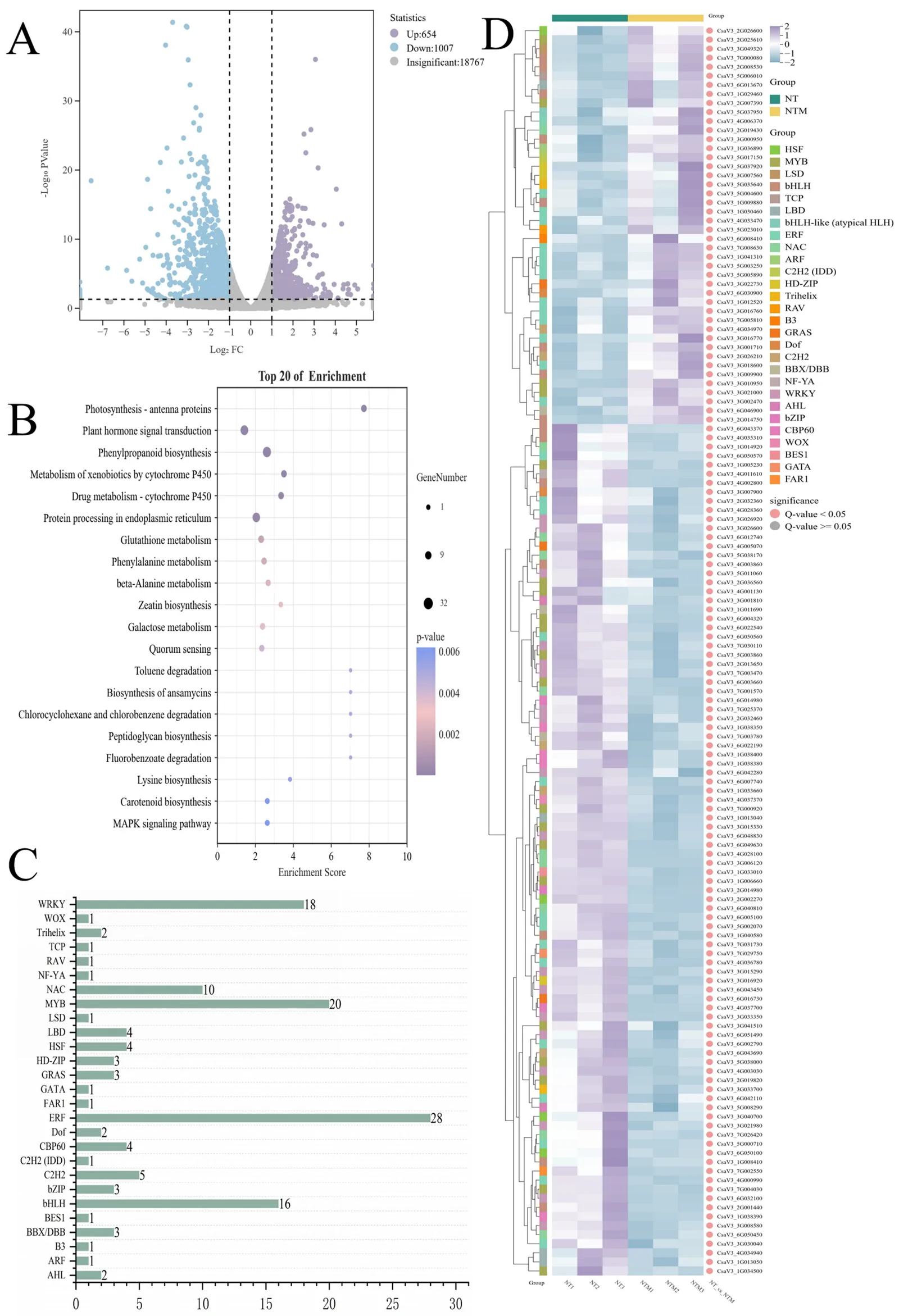
Differentially expressed gene analysis of cucumber in response to melatonin treatment. NT, cucumber seedlings sprayed with distilled water and maintained at 25 °C for 12 h; NTM, seedlings sprayed with 75 μmol L^−1^ melatonin and maintained at 25 °C for 12 h. In the volcano plot, each point represents a gene. Blue and pink points indicate significantly downregulated and upregulated genes, respectively, whereas gray points represent genes without significant differential expression. The x-axis represents the change in gene expression (log_2_FC), with larger absolute log_2_FC values indicating greater differences in relative gene expression between the two treatments. Differentially expressed genes (DEGs) were identified using the thresholds |log_2_FC| > 1 and *p* < 0.05. In the KEGG enrichment bubble plot, the x-axis represents the enrichment factor, calculated as the ratio of the number of DEGs assigned to a given pathway to the total number of genes annotated to that pathway. A larger enrichment factor indicates a higher degree of pathway enrichment. The y-axis shows the KEGG pathway names. Bubble color represents the *p* value, with increasingly purple colors indicating greater statistical significance, whereas bubble size represents the number of DEGs enriched in each pathway. In the transcription factor expression heatmap, the x-axis represents the samples and the y-axis represents gene IDs. Red dots preceding the gene IDs indicate transcription factors that were significantly differentially expressed between the distilled-water and 75 μmol L^−1^ melatonin treatments. Colored bars at the top indicate sample groups, whereas colored bars on the left indicate different transcription factor families. (**A**) Volcano plot of DEGs following melatonin treatment. (**B**) KEGG enrichment analysis of DEGs following melatonin treatment. (**C**) Distribution of transcription factors identified among melatonin-responsive DEGs by family. (**D**) Expression patterns of transcription factors identified following melatonin treatment.

A total of 1661 DEGs were identified in cucumber following exogenous melatonin treatment, including 654 upregulated and 1007 downregulated genes (Figure 4A). Notably, the number of downregulated genes was substantially greater than that of upregulated genes, indicating that melatonin treatment was predominantly associated with transcriptional repression under the experimental conditions.

KEGG enrichment analysis of the melatonin-responsive DEGs showed that the most significantly enriched pathways were Plant hormone signal transduction, Phenylpropanoid biosynthesis, and Protein processing in endoplasmic reticulum (Figure 4B). These enrichment patterns were similar to those observed under temperature stress, suggesting that melatonin treatment and temperature stress may affect several overlapping biological processes.

Among the melatonin-responsive DEGs, 138 transcription factors (TFs) were identified. These TFs were mainly assigned to the ERF, MYB, WRKY, and bHLH families (Figure 4C). Cluster analysis further revealed pronounced differences in the expression patterns of these TFs, with substantially more TFs showing reduced expression than increased expression. This overall trend was consistent with the distribution of up- and downregulated genes observed in the volcano plot analysis (Figure 4D).

### 2.3. Analysis of Genes Responding to Melatonin and Temperature Stress

**Figure 5:**
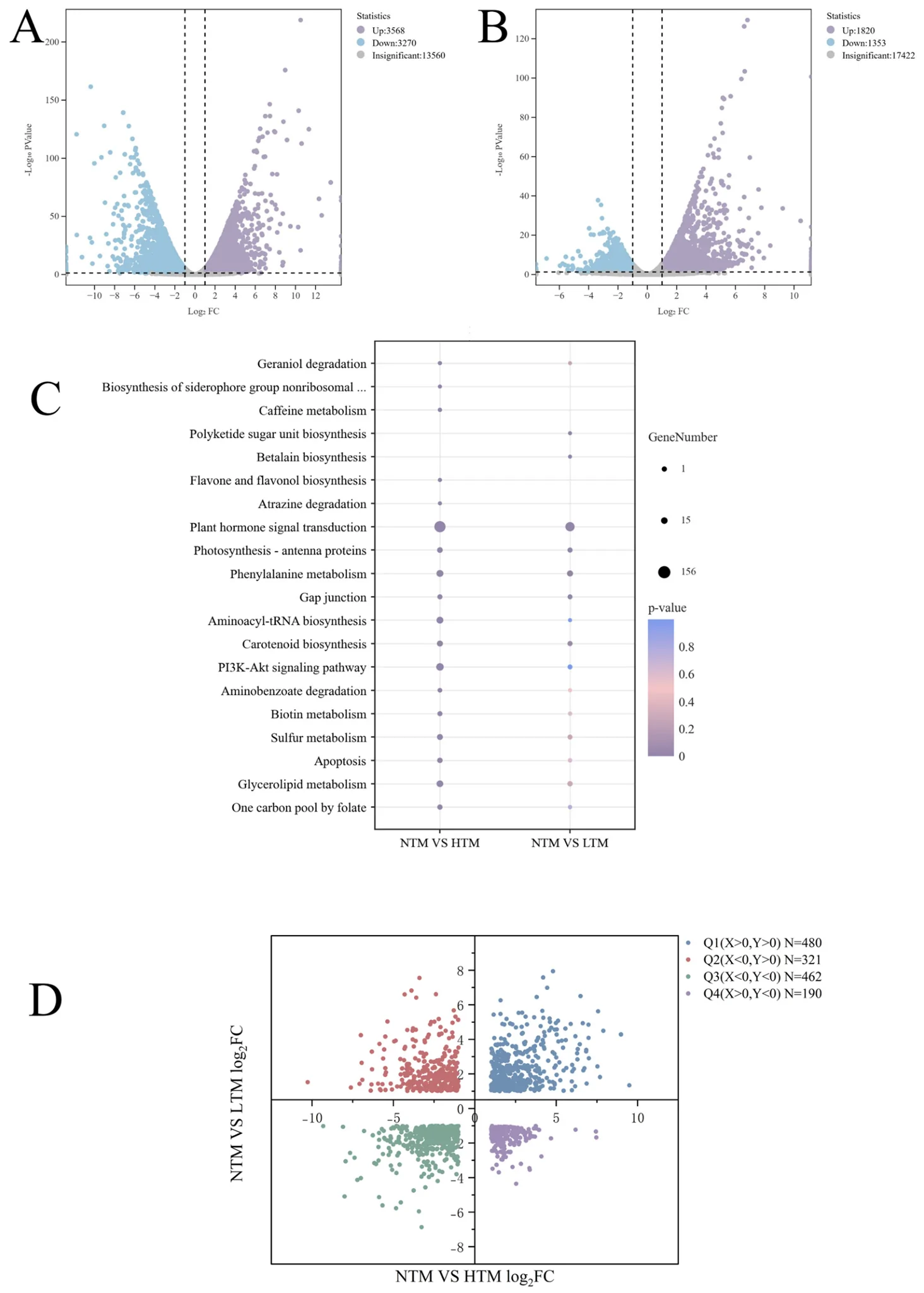
Differentially expressed gene analysis of cucumber under heat and cold stress following melatonin treatment. NTM, cucumber seedlings sprayed with 75 μmol L^−1^ melatonin and maintained at 25 °C for 12 h; HTM, seedlings sprayed with 75 μmol L^−1^ melatonin and exposed to 40 °C for 12 h; LTM, seedlings sprayed with 75 μmol L^−1^ melatonin and exposed to 4 °C for 12 h. In the volcano plots, each point represents a gene. Blue and pink points indicate significantly downregulated and upregulated genes, respectively, whereas gray points represent genes without significant differential expression. The x-axis represents the change in gene expression (log_2_FC), with larger absolute log_2_FC values indicating greater differences in relative gene expression between the two groups. Differentially expressed genes (DEGs) were identified using the thresholds |log_2_FC| > 1 and *p* < 0.05. In the KEGG enrichment bubble plots, the x-axis represents the enrichment factor, calculated as the ratio of the number of DEGs assigned to a given pathway to the total number of genes annotated to that pathway. A larger enrichment factor indicates a higher degree of pathway enrichment. The y-axis shows the KEGG pathway names. Bubble color represents the *p* value, with increasingly purple colors indicating greater statistical significance, whereas bubble size represents the number of DEGs enriched in each pathway.In the comparative analysis of DEGs, the x-axis represents the log_2_FC values under heat stress and the y-axis represents those under cold stress. Q1 (blue) represents genes upregulated under both heat and cold stress; Q2 (red) represents genes downregulated under heat stress but upregulated under cold stress; Q3 (green) represents genes downregulated under both heat and cold stress; and Q4 (purple) represents genes upregulated under heat stress but downregulated under cold stress. (**A**) Volcano plot of DEGs under heat stress following treatment with 75 μmol L^−1^ melatonin. (**B**) Volcano plot of DEGs under cold stress following treatment with 75 μmol L^−1^ melatonin. (**C**) KEGG enrichment analysis of DEGs under heat and cold stress following melatonin treatment. (**D**) Comparative analysis of DEGs shared between heat and cold stress following melatonin treatment.

A total of 6838 DEGs were identified in cucumber under heat stress following melatonin treatment, including 3568 upregulated and 3270 downregulated genes (Figure 5A). Under cold stress following melatonin treatment, 3173 DEGs were detected, of which 1820 were upregulated and 1353 were downregulated (Figure 5B). Thus, under melatonin-treated conditions, heat stress induced a larger transcriptional response than cold stress. This pattern was consistent with that observed in cucumber subjected to temperature stress without melatonin treatment.

KEGG enrichment analysis showed that Plant hormone signal transduction was the most significantly enriched pathway under both heat and cold stress following melatonin treatment. This was followed by Phenylpropanoid biosynthesis and Protein processing in endoplasmic reticulum (Figure 5C).

Comparative analysis of the DEGs responsive to heat and cold stress under melatonin-treated conditions identified 1453 genes shared between the two temperature treatments. Among these, 480 genes were upregulated under both heat and cold stress, whereas 462 genes were downregulated under both conditions. In addition, 321 genes were downregulated under heat stress but upregulated under cold stress, while 190 genes were upregulated under heat stress but downregulated under cold stress (Figure 5D).

### 2.4. Integrated Analysis of Transcriptomic Results from Different Experimental Groups

**Figure 6:**
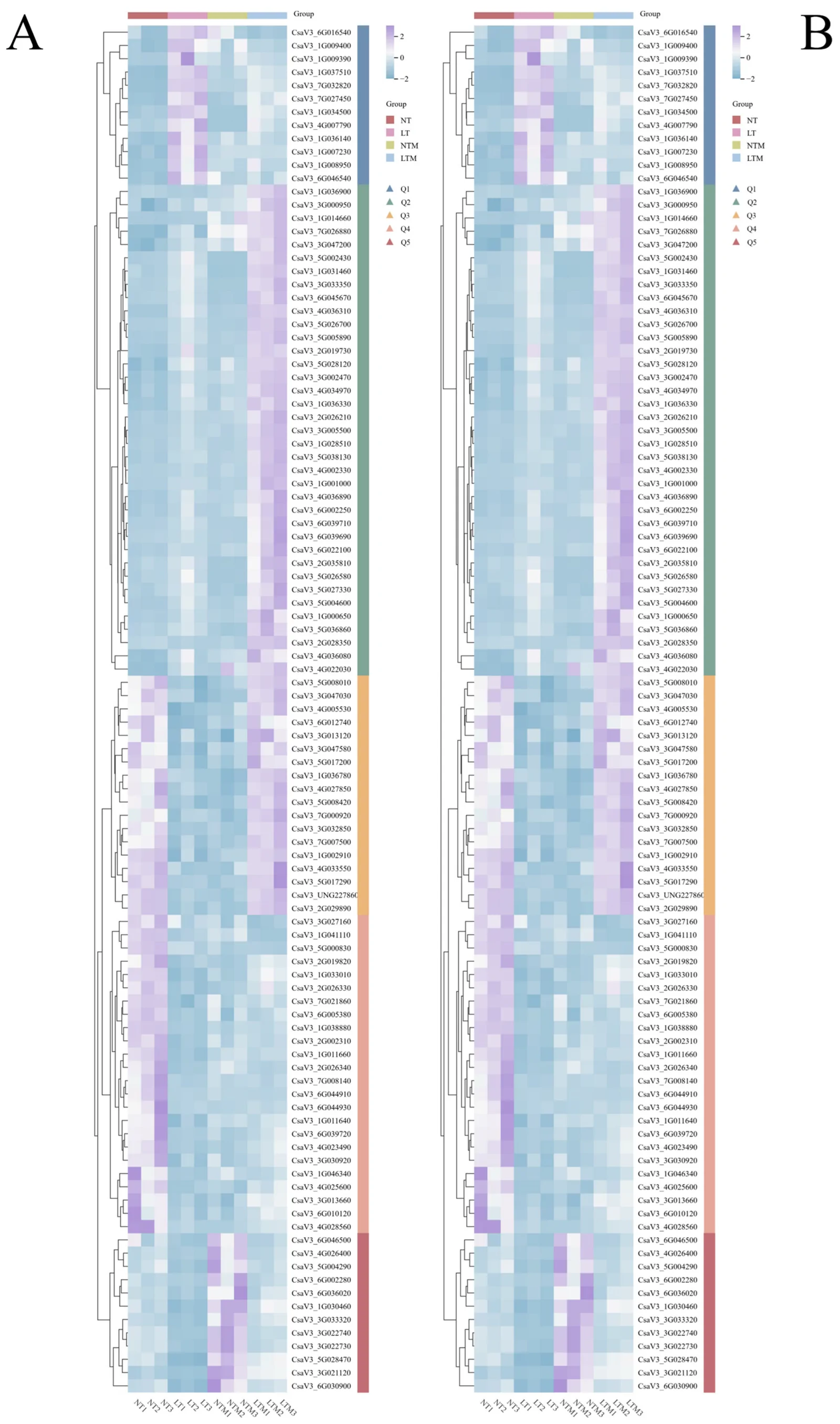

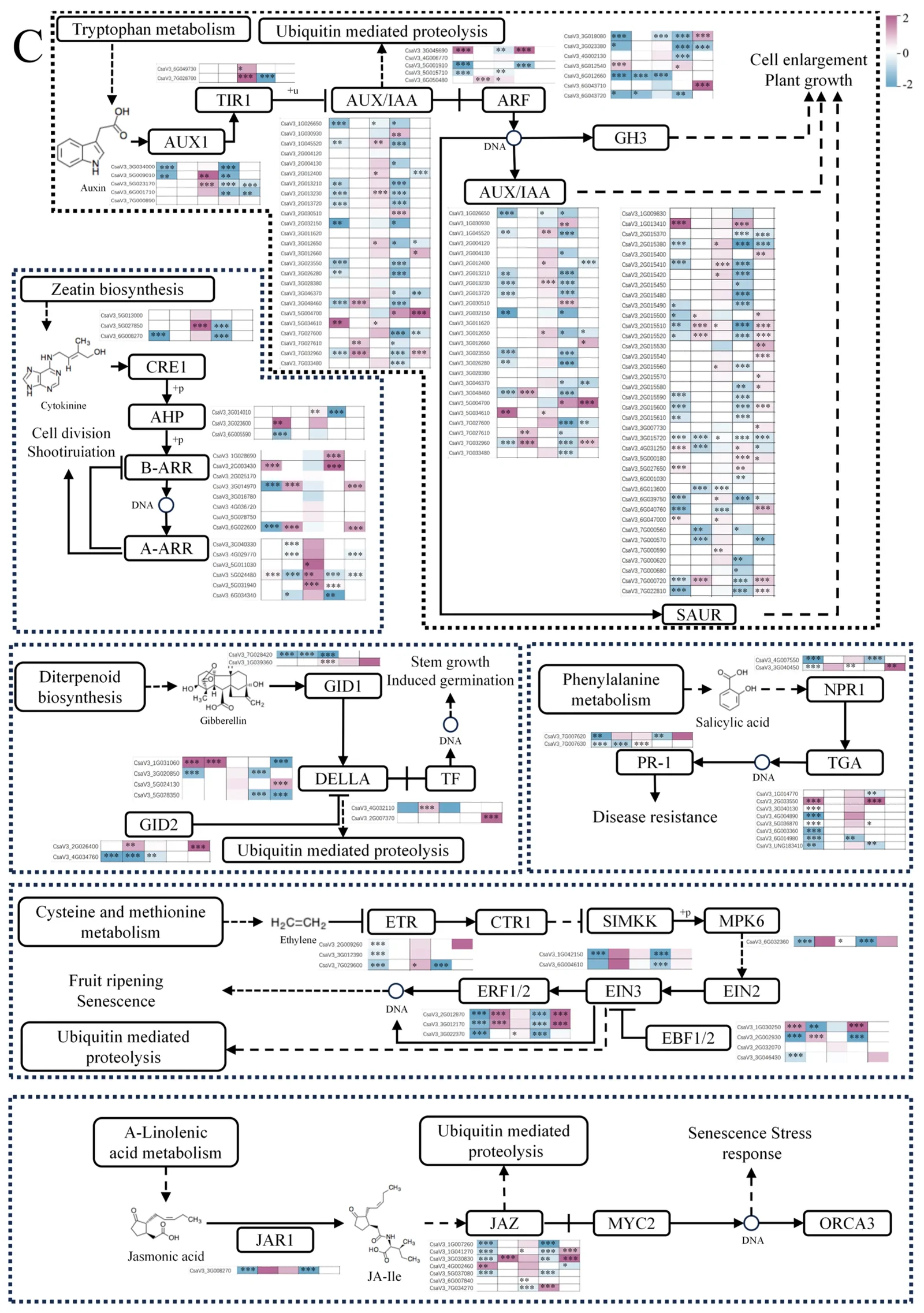

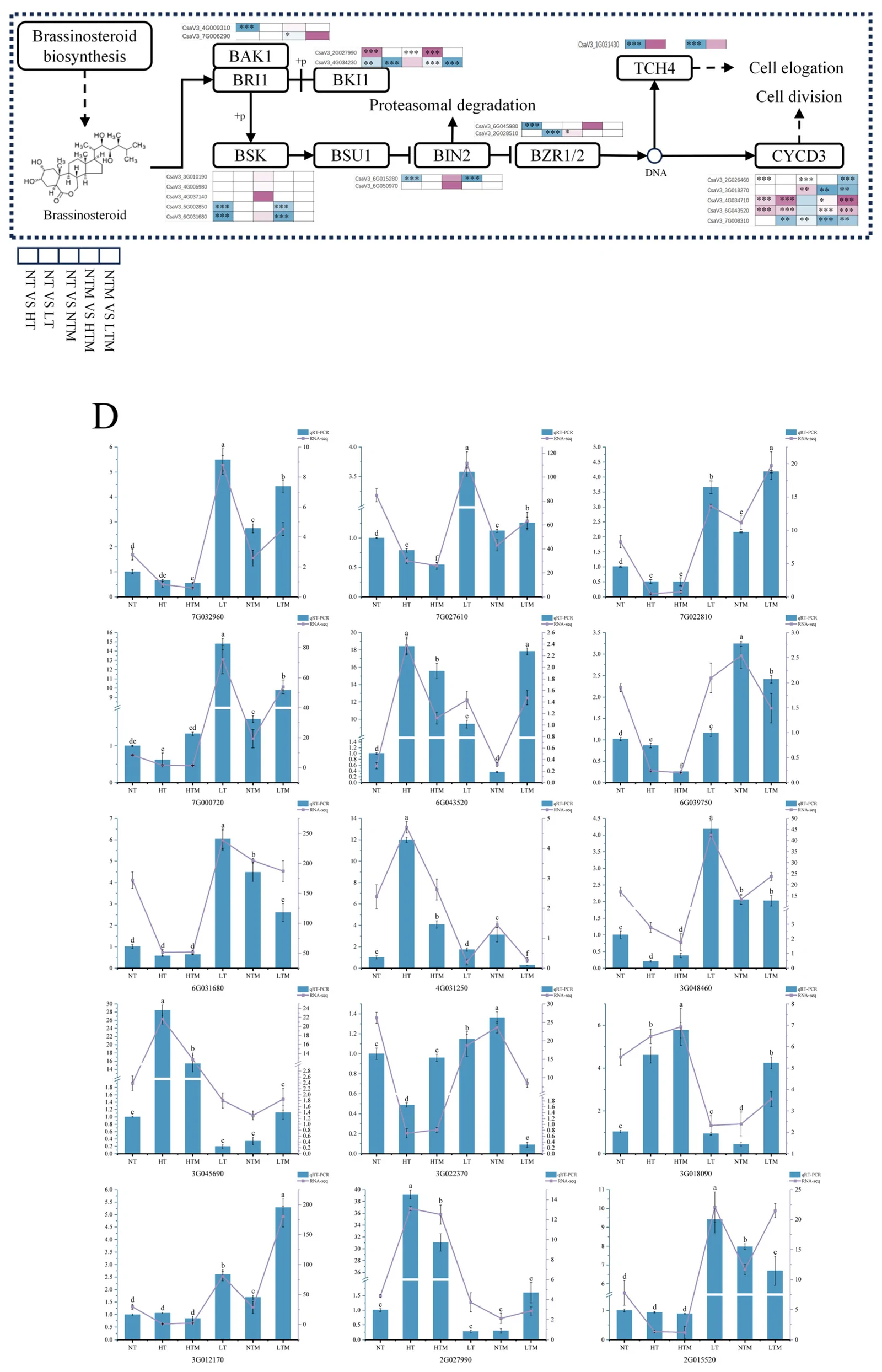
Integrated analysis of transcriptomic data. (**A**) Expression-pattern analysis of differentially expressed genes under heat-stress conditions. The x-axis represents the different treatment groups: NT, cucumber seedlings sprayed with distilled water and maintained at 25 °C for 12 h; HT, seedlings sprayed with distilled water and exposed to 40 °C for 12 h; NTM, seedlings sprayed with 75 μmol L^−1^ melatonin and maintained at 25 °C for 12 h; and HTM, seedlings sprayed with 75 μmol L^−1^ melatonin and exposed to 40 °C for 12 h. The y-axis represents gene IDs. Expression patterns were visualized using FPKM values. Purple indicates relatively higher gene expression, whereas blue indicates relatively lower expression. Triangles of different colors denote distinct expression-pattern clusters. (**B**) Expression-pattern analysis of differentially expressed genes under cold-stress conditions. The x-axis represents the different treatment groups: NT, cucumber seedlings sprayed with distilled water and maintained at 25 °C for 12 h; LT, seedlings sprayed with distilled water and exposed to 4 °C for 12 h; NTM, seedlings sprayed with 75 μmol L^−1^ melatonin and maintained at 25 °C for 12 h; and LTM, seedlings sprayed with 75 μmol L^−1^ melatonin and exposed to 4 °C for 12 h. The y-axis represents gene IDs. Expression patterns were visualized using FPKM values. Purple indicates relatively higher gene expression, whereas blue indicates relatively lower expression. Triangles of different colors denote distinct expression-pattern clusters. (**C**) Analysis of the *Plant hormone signal transduction* pathway. Heatmaps were generated using log_2_FC values. Statistical significance is indicated by asterisks: *p* < 0.05 (*), *p* < 0.01 (**), and *p* < 0.001 (***). (**D**) Integrated comparison of qRT-PCR and RNA-seq results. Relative gene expression levels determined by qRT-PCR were calculated using the 2^−ΔΔCt^ method. Bars represent qRT-PCR results, whereas lines represent RNA-seq expression profiles. The employment of lowercase letters serves to denote the presence of statistically significant disparities between the data collected for the distinct experimental groups.

Temperature stress severely restricts plant growth and development. Melatonin is an endogenous plant biomolecule that has been widely associated with enhanced tolerance to adverse environmental conditions. To investigate transcriptional responses shared between temperature stress and melatonin treatment, genes responsive to heat stress, cold stress, and melatonin were screened and subjected to expression-pattern clustering. Two major expression patterns were observed among genes jointly responsive to temperature stress and melatonin. Under heat stress, genes in the blue module showed a moderate increase in expression, whereas their expression was further elevated when heat stress was applied following melatonin treatment. By contrast, genes in the green module were strongly induced by heat stress but showed relatively low expression when heat stress was imposed after melatonin treatment (Figure 6A). These contrasting expression patterns suggest that genes in the blue and green modules may be associated with melatonin-mediated adjustment of heat-stress responses in cucumber. Under cold stress, genes in the blue module were induced by low temperature but showed markedly reduced expression when cold stress was applied following melatonin treatment. Genes in the green module exhibited the opposite pattern: their expression decreased under cold stress but increased when cold stress was imposed after melatonin treatment (Figure 6B). These results suggest that the two modules may also participate in the transcriptional regulation associated with cucumber responses to cold stress.

Based on the transcriptomic comparisons among the different treatment groups, differentially expressed genes were further mapped to the Plant hormone signal transduction pathway to examine coordinated changes in hormone-related signaling following melatonin treatment (Figure 6C). Integrated analysis of this pathway revealed distinct hormone-related transcriptional patterns under heat and cold stress. Heat stress predominantly activated stress-responsive modules involving ethylene–MAPK–ERF, SA–NPR1/TGA–PR-1, and JA–JAR1/JAZ signaling. Genes associated with cell expansion, including small auxin-up RNA (SAUR) and xyloglucan endotransglucosylase/hydrolase 22 (TCH4), were also induced, suggesting that cucumber simultaneously activates defense responses and growth-related transcriptional reprogramming under high-temperature conditions. In contrast, the ethylene–ERF and SAUR–TCH4 modules were generally suppressed or exhibited gene-specific responses under cold stress. These changes were accompanied by complex transcriptional reprogramming of cytokinin, gibberellin, auxin, and brassinosteroid signaling components. Thus, cucumber does not appear to respond to 4 °C and 40 °C through an identical hormone regulatory network but instead displays two distinct, temperature-biased transcriptional programs.

Comparison of NT and NTM further showed that treatment with 75 μmol L^−1^ melatonin at 25 °C altered the expression of several hormone signaling-related genes, including members of the *AUX/IAA*, *A-ARR*, *GID1*, *BKI1*/*BIN2*, and *CYCD3* groups. These findings suggest that melatonin can alter the expression of hormone signaling-related genes even under normal conditions. It may also influence how these signaling pathways respond when plants are subsequently exposed to temperature stress. On this basis, we propose that melatonin may contribute to temperature adaptation through a combination of preconditioning and selective remodeling of hormone-associated signaling networks rather than simply suppressing stress-induced responses.

To validate the RNA sequencing (RNA-seq) results, genes from the Plant hormone signal transduction pathway were selected for quantitative real-time polymerase chain reaction (qRT-PCR) analysis. The expression trends obtained by qRT-PCR were generally consistent with those observed in the RNA-seq dataset based on Fragments Per Kilobase of transcript per Million mapped reads (FPKM) values (Figure 6D), providing independent support for the reliability of the transcriptomic expression patterns.

## 3. Discussion

Temperature fluctuations can substantially affect plant growth and cellular homeostasis by altering key physiological, metabolic, and molecular processes involved in normal development and stress adaptation. Low temperature can restrict photosynthesis in thermophilic species and activate cold-responsive transcriptional networks, whereas high temperature can disrupt protein homeostasis and redox balance and alter the expression of heat-responsive genes [2,3,28]. In the present study, exposure of cucumber seedlings to either 40 °C or 4 °C for 12 h caused varying degrees of wilting and induced extensive transcriptional changes. A total of 7488 differentially expressed genes (DEGs) were identified under heat stress, compared with 3400 under cold stress, indicating that, under the same treatment duration and DEG selection criteria, heat stress elicited a broader transcriptional response. Among these DEGs, 1757 responded to both heat and cold stress. Of these, 1122 showed the same direction of expression change under the two conditions, whereas 635 exhibited opposite expression patterns. These results indicate that cucumber responses to high and low temperatures involve both shared and temperature-specific regulatory components. Previous studies in cucumber have shown that natural variation in *CsSGR* and the *CsGPA1*–*CsCOR413PM2*–Ca^2+^-associated signaling process contribute to cold tolerance [7,9,11], supporting the existence of specific genetic regulatory mechanisms underlying the cucumber cold response. In the present study, DEGs induced by both high and low temperatures were enriched in pathways related to plant hormone signal transduction, phenylpropanoid biosynthesis, and protein processing in the endoplasmic reticulum, suggesting that these processes may represent common components of the cucumber response to abnormal temperatures.

Foliar application of 75 μmol L^−1^ melatonin partially alleviated the wilting phenotype of cucumber seedlings under both heat and cold stress, suggesting that exogenous melatonin can mitigate, to some extent, the damage caused by temperature stress. Melatonin is widely distributed in plants and has been implicated in the regulation of redox homeostasis, photosynthesis, antioxidant defense, and stress responses, including the modulation of antioxidant enzymes such as superoxide dismutase (SOD), catalase (CAT), peroxidase (POD), and ascorbate peroxidase (APX). [21,22,29,30]. In cucumber, Amin et al. reported that melatonin alleviated growth inhibition, photosynthetic decline, and oxidative damage caused by combined low-temperature and high-humidity stress [23]. Melatonin has also been associated with enhanced tolerance to low or high temperature in pepper and apple [25,26]. These findings are consistent with the phenotype of alleviated temperature stress observed in this study following the spraying of cucumber seedlings with a melatonin solution. Plant hormone signal transduction was significantly enriched in comparisons involving heat stress, cold stress, and melatonin treatment and therefore represented one of the most consistent transcriptomic features of this study. Integrated pathway analysis showed that heat stress caused pronounced expression changes in ethylene-associated MAPK–ERF signaling, SA-associated NPR1/TGA–PR-1 signaling, and JA-associated JAR1/JAZ signaling. In contrast, under cold stress, ethylene-related genes and components associated with auxin, cytokinin, gibberellin, and brassinosteroid signaling exhibited distinct response patterns. Thus, although both high and low temperatures affected hormone-related signaling, the direction and composition of these responses differed substantially. Notably, melatonin treatment under normal temperature conditions already altered the expression of genes related to *AUX*/*IAA*, *A-ARR*, *GID1*, *BKI1*/*BIN2*, and *CYCD3*. Melatonin treatment further modified the expression patterns of multiple hormone-signaling components during subsequent temperature stress. These observations are consistent with the current view that melatonin interacts extensively with endogenous plant hormone networks [21,22,24,25,31,32,33]. For example, Li et al. observed that melatonin treatment in pepper was accompanied by changes in auxin, gibberellin, and cytokinin metabolism [25], whereas Gao et al. showed in apple that *MdASMT9*-mediated melatonin accumulation influenced ABA biosynthetic genes through a regulatory pathway involving *MdWRKY33* [26].

Integrated clustering across the different treatment groups further showed that melatonin did not cause a uniform increase or decrease in the expression of all temperature-responsive genes. Under heat stress, one subset of heat-induced genes showed even higher expression after melatonin pretreatment, whereas another subset of heat-induced genes displayed relatively lower expression in the presence of melatonin. A similarly divergent pattern was observed under cold stress. Some cold-induced genes were expressed at lower levels after melatonin treatment, whereas other genes that were initially repressed by cold stress showed increased expression following melatonin application. These results indicate that melatonin modifies temperature-stress transcriptional responses in a gene- and expression-pattern-dependent manner. Melatonin is generally considered a multifunctional plant signaling molecule rather than a regulator acting through a single pathway [21,22]. Accordingly, our results suggest that exogenous melatonin may reshape the magnitude and direction of selected temperature-responsive transcriptional programs rather than globally enhancing or suppressing the stress response.

Among the DEGs responsive to melatonin under normal temperature conditions, 138 transcription factors were identified, predominantly from the ERF, MYB, WRKY, and bHLH families, and these genes displayed pronounced differential expression. Plant transcription factors are key regulatory nodes that connect environmental signal perception with downstream gene expression. Members of the ERF, WRKY, MYB, and bHLH families have been widely implicated in plant responses to abiotic stress. Under heat stress, transcription factors from multiple families collectively form complex regulatory networks [28,34,35], whereas the ICE–CBF module and its associated transcriptional regulators represent central components of the classical cold-response pathway [3,36,37,38]. In cucumber, *CsCBF1* can directly regulate *CsSGR*, and *CsGPA1*-associated signaling has been reported to influence *CsICE*–*CsCBF* expression [7,9]. In addition, studies in apple have provided evidence for functional interactions between melatonin-related pathways and WRKY transcription factors [26,39]. Together, these findings support an important role for transcriptional regulation in cucumber temperature-stress responses.

Taken together, the phenotypic and transcriptomic data suggest that exogenous melatonin at 75 μmol L^−1^ can alter the basal expression state of a subset of genes, particularly those associated with hormone signaling, even under normal temperature conditions. During subsequent exposure to heat or cold stress, melatonin-treated plants exhibited transcriptional responses distinct from those of untreated plants, accompanied by a less severe wilting phenotype. We therefore propose that melatonin may contribute to temperature-stress adaptation in cucumber seedlings by first modifying the basal transcriptional state and subsequently reshaping selected hormone-signaling components, transcription factors, and other stress-responsive genes during temperature stress. This interpretation is broadly consistent with previous studies describing melatonin as a multifunctional regulator of plant stress responses [21,22,23,25,26].

## 4. Materials and Methods

### 4.1. Growth and Treatment of Cucumis sativus Material

The cucumber inbred line ‘Xintaimici’ was utilised in this study. Plants were grown in a greenhouse at Southwest Forestry University using a substrate consisting of peat and vermiculite at a ratio of 4:1 (*v*/*v*). In addition, other standard cultivation practices were followed throughout the experiment to maintain plant health, as recommended by Liang [40].

Upon attaining full expansion of their second true leaf, seedlings exhibiting uniform growth were subjected to the following treatments: (1) leaves were sprayed with distilled water, followed by exposure to 4 °C for 12 h (LT); (2) leaves were sprayed with distilled water, followed by exposure to 40 °C for 12 h (HT); (3) leaves were sprayed with 75 μmol L^−1^ melatonin and maintained at 25 °C for 12 h (NTM); (4) leaves were sprayed with 75 μmol L^−1^ melatonin, followed by exposure to 4 °C for 12 h (LTM); (5) leaves were sprayed with 75 μmol L^−1^ melatonin, followed by exposure to 40 °C for 12 h (HTM); and (6) leaves were sprayed with distilled water and maintained at 25 °C for 12 h (NT).

After treatment, the second leaves (from the bottom) were collected and gently rinsed with phosphate-buffered saline (PBS) to remove residues from the leaf surface. The samples were immediately frozen in liquid nitrogen and subsequently stored at −80 °C until further analysis.

### 4.2. Transcriptome Sequencing and Data Quality Control

Total RNA was extracted from cucumber leaves collected from each treatment group using the CTAB-PBIOZOL method. RNA quality and concentration were subsequently assessed using a Qubit fluorometer (Thermo Fisher Scientific Inc., MA, USA) and a Qsep400 high-throughput biofragment analyzer (Vazyme Biotech Co., Ltd., Nanjing, China). RNA-seq libraries were constructed using the NEBNext Ultra™ RNA Library Prep Kit (New England Biolabs, Inc., Ipswich, MA, USA), with 1 μg of total RNA used for library preparation from each sample. After library construction, library concentration and fragment size distribution were evaluated using an Agilent 2100 Bioanalyzer (Agilent Technologies, Santa Clara, CA, USA). The effective library concentration was then accurately quantified by quantitative PCR (qPCR). Following quality control, individual libraries were pooled according to their effective concentrations and the desired sequencing depth. Sequencing was performed on an Illumina NovaSeq 6000 platform (Illumina Inc., San Diego, CA, USA) in paired-end 150-bp mode (PE150; 2 × 150 bp).

Raw sequencing reads were subjected to quality control using Trimmomatic. Adapter sequences and low-quality bases were removed to generate high-quality clean reads [41]. The clean reads were subsequently aligned to the cucumber reference genome available in CuGenDBv2 (accessed June 2026) using HISAT2 with default parameters [42]. The overall genome alignment rate was used as an indicator of sequencing and sample quality.

### 4.3. Quantification at the Gene Level, Screening for Differentially Expressed Genes and Functional Enrichment

The alignment results of clean reads against the reference genome were stored in binary alignment/map (BAM) format. Transcript abundance, expressed as fragments per kilobase of transcript per million mapped reads (FPKM) [43], and read counts were quantified using Bowtie2 [44] and eXpress [45]. The numbers of reads assigned to individual protein-coding transcripts in each sample were obtained using eXpress. Read-count data were normalized using the estimateSizeFactors function implemented in the DESeq R package [46]. The nbinomTest function was subsequently used to calculate p values and fold changes for pairwise comparisons between treatment groups. Transcripts with *p* < 0.05 and |log_2_FC| > 1 were considered differentially expressed. These differentially expressed transcripts were further subjected to Kyoto Encyclopedia of Genes and Genomes (KEGG) enrichment analysis [47] to identify the major biological functions and pathways associated with the observed transcriptional changes.

### 4.4. Quantitative Analysis by qRT-PCR

Based on the integrated transcriptomic analysis, a subset of genes involved in the Plant hormone signal transduction pathway was randomly selected for validation by quantitative reverse transcription PCR (qRT-PCR). Total RNA was extracted using the FastPure Plant Total RNA Kit (Vazyme Biotech Co., Ltd., Nanjing, China), and first-strand cDNA was synthesized using the All-in-One 5× RT MasterMix Kit (Applied Biological Materials Inc., Shanghai, China). Gene-specific primers for qRT-PCR were designed and checked using the BLAST tool available from the National Center for Biotechnology Information (NCBI; https://blast.ncbi.nlm.nih.gov/Blast.cgi, accessed on 20 June 2026), and the primer sequences are listed in Table 2. The *Actin* gene was used as the internal reference gene. Relative transcript abundance was calculated using the 2^−ΔΔCt^ method [48]. Amplification was conducted utilising a LightCycler® 480II system (Roche, Penzberg, Germany).

## 5. Conclusions

By analysing transcriptomic data from cucumber plants subjected to different treatments, the study systematically elucidated the distinct responses of cucumber to heat and cold stress and investigated how melatonin enhances stress tolerance through pre-adaptation and selective reprogramming of signalling pathways. Nevertheless, the underlying mechanisms remain to be fully elucidated and warrant further investigation. Overall, these findings provide valuable insights into the role of melatonin in enhancing plant resilience to temperature stress.

## Figures and Tables

**Table 1:** Transcriptome quality control table.

Sample Group	Clean Bases	GC(%)	Q30 (%)	Reads Mapped(%)
NT	7.19–7.30 G	43.76–43.83	93.51–95.66	98.27–98.34
HT	7.27–7.35 G	43.73–43.85	93.68–95.54	98.34–98.42
LT	7.10–7.46 G	43.13–43.34	95.09–95.66	98.18–98.39
NTM	6.98–7.54 G	42.36–44.47	93.52–95.54	97.71–98.64
HTM	6.99–7.29 G	43.18–43.73	93.57–93.67	98.29–98.41
LTM	7.00–7.55 G	43.09–43.77	93.63–94.81	98.29–98.38

**Table 2:** The primer sequences used in this research.

Gene ID	Forward Primer (5′→3′)	Reverse Primer (5′→3′)
1G030250	TACCGGCTTTGGTGCTGAAA	CTATGAGCCCACGATCCGAG
1G031430	AAGGCAAAGGCGACAGAGAA	TTCTGTACGCCGCTGTGAAT
2G002930	CGAGGGATGACCGATGTGAG	CACCTCCACAGGTTCTCGAC
2G003430	ACATGAGCAGCTCTTCGACC	ATGAGCCGAGAGATTGTGCG
2G012870	AACTCCGATCTCTCACCCGA	TTCTCCGAGGGAATCGACCT
2G015500	TGTTCCTAAAGGTCACATCGCA	TCCTGTTGGGTGGTTGAATCC
2G015510	TGGTTCCTCATGCCAAGCA	TCTCCCACATAAACGGCAACA
2G015520	CAGCGCCAAGCAAATTCTCAA	ACACCGGAATGTGGCCTTTG
2G027990	AGCCAAAACCTCACCTTCCT	CATGGAAACTGGCGCTGAGA
3G012170	CGGAGAAGAAAGCCCGAAGA	TAAACTATCACCGCTGCCCC
3G015720	GGTGTGCGTGGACTGTGTAG	GCCTCACTTTCTTCCGCCT
3G018080	ACCATTTCAAGACCCGACCC	GGTCCACGAGTTTTGGGGAT
3G022370	CGGTGGAAATGGTGAGGGAA	CTACCACCGGCGAACATCC
3G045690	GCCGGTCCTCTTGTTTCCTT	ATGTGGCGAAAAGTCCAGGT
3G048460	TTCCAACCCCACAAGCTGAT	TCGGGGAACAGCGAAGTTTT
4G031250	TGGTTAGCTGTGGAAGTAGGG	GTCGTCGACTGAGCATGGAA
4G034230	CTTCTTCGCCTCGTTCCTCC	GCTCGGTAATCTCGCATCCT
4G034710	ACTCACCACAACACTTCCCT	GGTTGGAACTTCGGTTGGGA
5G002850	TTGGATGAAGCAAGGGCTGT	CGACGTGCAAATACCGTTGG
5G024480	CGAACGCCTCCTCAAAACCT	TGGAGTCCGTTTGCTGATCG
6G001710	TGGCAACCTCCAAACAGGG	ACATTTCCACAGCATGCCAC
6G012660	AGCTCGTATTGCTGACGGTG	TGCAATAACGGTACAGCCCA
6G031680	TGCAAGGATGGATCTCACGG	CAAATCACCACGCTTCCTCG
6G039750	GCGCGATCGACAACACTAAG	TGCTGCCAATGGGTTTTGTG
6G043520	GTGGAGGAGACCCAAGTTCC	TGGCGCAATCTCACTGATGG
6G043720	TTCAACTCCTCTTGGACCGC	CAGTGTGAGCGTTTTGGGTG
7G000720	CGTCGTTTTCCGATGTTCCG	TGGCACAACAAATCGCTTCC
7G022810	TCGTGTTGCCGATTCCTCTC	AGGATTCGAGAACGGCGAAG
7G027610	TCTCAACTTCCAAGCCACCG	CGACTTTGATTCGGTGCGAC
7G032960	AGAGTCGTCCTCGTTGTTGC	TCCCTGTCCATCCATTTTCCC
*CsActin*	AGAAGATCTGGCATCACA	TCCAATCCAGACACTGTACT

## Data Availability

All data are available in the main text. The transcriptome sequence data have been uploaded to the NCBI database, BioProject ID: PRJNA1084249; access link: https://www.ncbi.nlm.nih.gov/bioproject/?term=PRJNA1084249 (access date: 20 June 2026).
